# Article Watch: December, 2025

**DOI:** 10.7171/001c.159586

**Published:** 2025-12-12

**Authors:** Clive Slaughter

**Affiliations:** 1 Augusta University - University of Georgia at Athens Medical Partnership

**Keywords:** literature, research article, current article

## Abstract

This column highlights recently published articles that are of interest to the readership of this publication. We encourage ABRF members to forward information on articles they feel are important and useful to Clive Slaughter, AU-UGA Medical Partnership, 1425 Prince Avenue, Athens GA 30606. Tel; (706) 713-2216: Fax; (706) 713-2221: Email; cslaught@uga.edu or to any member of the editorial board. Article summaries reflect the reviewer’s opinions and not necessarily those of the Association.

## NUCLEIC ACID SEQUENCING AND GENOTYPING

Chen Y, Davidson N M, Wan Y K, Yao F, Su Y, Gamaarachchi H, Sim A, Patel H, Low H M, Hendra C, Wratten L, Hakkaart C, Sawyer C, Iakovleva V, Lee P L, Xin L, Ng H E V, Loo J M, Ong X, Ng H Q A, Wang J, Koh W Q C, Poon S Y P, Stanojevic D, Tran H-D, Lim K H E, Toh S Y, Ewels P A, Ng H-H, Iyer N G, Thiery A, Chng W J, Chen L, DasGupta R, Sikic M, Chan Y-S, Tan B O P, Wan Y, Tam W L, Yu Q, Khor C C, Wüstefeld T, Lezhava A, Pratanwanich P N, Love M I, Goh W S S, Ng S B, Oshlack A, Iyer N G, Yu Q, Göke J, consortium S G-N. A systematic benchmark of Nanopore long-read RNA sequencing for transcript-level analysis in human cell lines. *Nature Methods* 22;2025:801-812.

Although the total RNA expressed by a gene is generally regarded as an adequate measure of the gene’s activity, physiologic importance may be also be ascribed to which RNA isoforms are expressed, and in what relative quantities. Short-read sequencing is expected to diminish the accuracy with which alternate RNA isoforms can be measured. Chen *et al.* here report the results of the Singapore Nanopore Expression project that benchmarks comparative advantages of the various long-read sequencing methods currently available for isoform expression analysis, including IsoSeq from Pacific Biosciences (Menlo Park, CA); and, from Oxford Nanopore Technologies (Oxford, U.K.), long-read sequencing of PCR-amplified cDNA, sequencing of amplification-free cDNA, and direct RNA sequencing. The authors compare these protocols one with another and compare them with short-read RNA-seq, using multiple replicates per sample and a panel of human cell lines, including spike-in RNA standards. Although long-read methods avoid bias in measured abundance introduced by the fragmentation step used in short-read methods, different instances of bias in measured abundance are introduced by amplification in the methods that employ PCR. The authors confirm that fragmentation does affect expression estimates for some isoforms. In genes that exhibit complex splicing events involving multiple exons, they ascertain that exon skipping is the most common basis for alternate isoforms, followed by usage of alternative promoters and alternative last exons. The authors identify novel transcripts of low abundance in long-read datasets and demonstrate the advantage of long-read sequencing for identifying fusion genes. The database compiled in this study is publicly available as a resource for developing computational methods.

van der Sanden B, Neveling K, Shukor S, Gallagher M D, Lee J, Burke S L, Pennings M, van Beek R, Oorsprong M, Kater-Baats E, Kamping E, Tieleman A A, Voermans N C, Scheffer I E, Gecz J, Corbett M A, Vissers L E L M, Pang A W C, Hastie A, Kamsteeg E-J, Hoischen A. Optical genome mapping enables accurate testing of large repeat expansions. *Genome Research* 35;2025:810-823.

This work addresses the need for increased precision and convenience in characterizing the length of short tandem repeat sequences. Expansion of such sequences, either in the germline or in somatic tissues, is responsible for a variety of neuromuscular diseases. The authors seek better precision for measuring these than the conventional approaches of repeat-primed PCR or Southern blotting, and more accessible approaches than long-read genomic sequencing. They explore optical genomic mapping for this purpose. Using the Saphyr technology from Bionano Genomics Inc. (San Diego, CA), ultra-high molecular weight DNA is isolated from cells, fluorescent labels are enzymatically attached to commonly occurring sequence motifs, and the resulting labeling patterns are imaged at high resolution on a chip after linearizing the unfragmented DNA. This approach provides genome-wide coverage that includes both members of homologous chromosome pairs. In addition to standard, manual, *de novo* data assembly, the authors contribute additional data analysis methods that provide orthogonal information. The results indicate that such optical genome mapping simultaneously provides accurate size measurements for multiple loci with short tandem repeats having alleles with expansions >300 bp. The methodology also permits detection of somatic instability in expansion length, but it doesn’t detect interruptions within repeat sequences that may protect against further expansion. The work is anticipated to contribute to diagnostic accuracy for repeat expansion disorders.

## MACROMOLECULAR CHARACTERIZATION

Fischer K, Lulla A, So T Y, Pereyra-Gerber P, Raybould M I J, Kohler T N, Yam-Puc J C, Kaminski T S, Hughes R, Pyeatt G L, Leiss-Maier F, Brear P, Matheson N J, Deane C M, Hyvönen M, Thaventhiran J E D, Hollfelder F. Rapid discovery of monoclonal antibodies by microfluidics-enabled FACS of single pathogen-specific antibody-secreting cells. *Nature Biotechnology* 43;2025:960-970.

Fischer *et al.* propose a streamlined, low-cost, high-throughput method for identifying and characterizing the individual antibody molecules that comprise a human (or mouse) humoral immune response. The authors are studying antibody-secreting B cells. Unlike the memory B cells that constitute the population usually selected for generating monoclonal antibodies, antibody-secreting cells express little or no immunoglobulin antigen receptors bound to their plasma membrane. The challenge addressed here is therefore to associate each secreted antibody with the cell clone that secreted it. The methodology deployed by the authors combines droplet microfluidics with conventional fluorescence-activated cell sorting (FACS) to isolate antigen-specific antibody-secreting cells for single-cell sequencing and recombinant antibody expression. Human peripheral blood mononuclear cells (or mouse bone marrow or spleen cells) are mixed with liquid agarose derivatized with benzylguanine. The benzylguanine groups will later be used for SNAP-tag derivatization with fused single-domain immunoglobulins that bind antibody light chain con-stant regions (either κ or λ) to capture secreted antibodies. But first, individual antibody-secreting cells in the liquid agarose at 37˚C are encapsulated into monodisperse water-in-oil emulsion droplets. These droplets are collected on ice, which solidifies the agarose by cooling. Demulsification of the droplets then creates a stable agarose hydrogel microcompartment for each cell into which it can secrete its antibody. Within these hydrogel beads, both the secreted antibody and the cell that secreted it can be characterized. The beads are stained, washed, and sorted by conventional FACS, and the single cells within them are sequenced to define the antibody, allowing subsequent recombinant expression. The system is capable of very high throughput, >10^7^ hydrogel beads per hour, enabling even rare antigen-specific clones to be identified in heterogenous cell populations. The authors identify monoclonal antibodies against SARS CoV2 that bind with high affinity (<1 pM) and high neutralizing capacity. The methodology is expected to facilitate the discovery of clinically useful antibodies and the characterization of the repertoire of antigen-specific antibodies elicited during immune responses to pathogens.

Bergonzo C, Grishaev A. Critical Assessment of RNA and DNA Structure Predictions via Artificial Intelligence: The Imitation Game. *Journal of Chemical Information and Modeling* 65;2025:3544-3554.

Using transformer neural networks, AlphaFold software has proven exceptionally successful in predicting protein structures based upon experimentally determined training data from the Protein Data Bank. The latest update, AlphaFold3, extends AlphaFold capabilities to include the prediction of the structure of complexes and of oligonucleotides. The present paper provides an assessment of the accuracy achieved by AlphaFold3 in predicting oligonucleotide structure. Accuracy is assessed by comparison with experimental data acquired using nuclear magnetic resonance (NMR) spectroscopy. Comparisons include the structural effects produced by introducing cations or sequence modifications. The results regarding AlphaFold3 accuracy are mixed. Good accuracy is exhibited with, for example, canonical base pairing, short RNA loops of common type, and tracts of DNA A structure, whereas poor accuracy is seen with less common or longer loops, increasingly complex structures, and perturbation by cations or non-canonical base pairings. The authors conclude that models of oligonucleotide structure based on artificial intelligence more closely mimic the training dataset than capture underlying structural reality. Supplementation of such models with experimental data is therefore recommended wherever possible.

## PROTEOMICS

Beimers W F, Overmyer K A, Sinitcyn P, Lancaster N M, Quarmby S T, Coon J J. Technical evaluation of plasma proteomics technologies. *Journal of Proteome Research* 24;2025:3074-3087.

Variation in the composition of the blood plasma proteome has long been studied to reveal information about whole-organism physiology and pathology. The wide dynamic range of abundance of plasma proteins (estimated at ~10^12^) has severely limited the breadth of analytical sampling. Beimers *et al.* compare the performance of four selected, recently developed methods that expand the depth of plasma proteome quantification. The Explore High Throughput (HT) technology from Olink Proteomics Inc. (Waltham, MA) employs a pair of antibodies for each of 5415 protein targets. When antibody pairs bind in close proximity, the attached DNA barcode sequences anneal and become amenable to amplification for quantification by high-throughput sequencing. The remaining three technologies are based on mass spectrometry: the ENRICHplus technology from PreOmics Inc (Planegg/Martinsried, Germany), the Proteograph XT technology from Seer Inc (Redwood City, CA), and the Mag-Net technology from ReSyn Biosciences (Edenvale, Gauteng, South Africa). These employ mixtures of paramagnetic nanoparticles with physical properties engineered to bind various subpopulations of proteins in so-called corona layers. The four technologies are compared to simple, unenriched plasma, and plasma precipitated with perchloric acid. The comparison includes proteome depth, reproducibility, linearity, tolerance to lipid interference, limit of detection/quantification, and cost/convenience. Sample preparation method is found to have a strong influence on sampling depth with MS-based technologies. Seer, the best performing MS technology, provides twice the depth of Olink, and overall better technical characteristics, but Olink has better sample throughput. Interestingly, there is little overlap between identifications using the MS-based technologies and Olink, indicating that the methodologies are complementary.

Suresh P S, Zhang Q. Comprehensive comparison of methods for isolation of extracellular vesicles from human plasma. *Journal of Proteome Research* 24;2025:2956-2967.

Extracellular vesicles, membrane-bound particles released by cells, are diverse in size (apoptotic bodies of 50-1000 nm, microvesicles of 100-1000 nm, and exosomes of 30-150 nm), and other physical characteristics. Because their composition and abundance reflect the state of their cells of origin, extracellular vesicles are of great interest as biomarkers of physiology and disease. Among the impediments to their study is the co-occurrence in blood plasma of lipoprotein particles with a similar span of sizes (chylomicrons of 200-600 nm, VLDL of 30-90 nm, LDL of 21-27 nm and HDL of 7-13 nm). Especially problematic is that lipoprotein particles occur at 10^3^–10^6^-fold higher levels.

Suresh & Zhang here compare the performance of nine methods commonly used to purify extracellular vesicles for characterization or exploitation. The methods separate particles by centrifugation, size-exclusion chromatography, polymer precipitation, electrostatic interaction, and affinity. The resulting particles are characterized by size and number (using nanoparticle tracking analysis), Western analysis, and proteomic analysis. Vesicle yield is challenged by restricting the starting material to 100 µL of plasma in each case to test sensitivity. The results indicate that tradeoffs in choosing a method for a given study are numerous and complex, but the reproducible behavior of the various techniques suggests that the present research benefits investigators making their choices.

Peters-Clarke T M, Liang Y, Mertz K L, Lee K W, Westphall M S, Hinkle J D, McAlister G C, Syka J E P, Kelly R T, Coon J J. Boosting the sensitivity of quantitative single-cell proteomics with infrared-tandem mass tags. *Journal of Proteome Research* 24;2025:1539-1548.

While the biological impact of single-cell proteomics has hitherto been poorer than that of single-cell transcriptomics, the potential importance of proteomics for understanding functional variation between individual cells remains very high. Peters-Clarke *et al.* here make substantial improvements in the analytical sensitivity and quantitative accuracy of single-cell proteomics at the level of mass spectrometric methodology. They adopt a tandem mass tag (TMT) approach using isobaric labeling, and employ carriers to boost the detection of MS1 precursor ions in a method known as Single-Cell ProtEomics by Mass Spectrometry (SCoPE-MS). They additionally minimize quantitative-ratio distortion resulting from co-isolation of background species in MS2 by existing methods for synchronous precursor selection (SPS) of multiple ions from MS2 spectra using a multinotch waveform for higher-energy collisional dissociation in a Q-Orbitrap-quadrupole ion trap for MS3 analysis. Here, they test the additional benefit of infrared TMT (IR-TMT), in which they use infrared multiphoton photoactivation and ion parking employing an RF parking waveform to maximize reporter generation and minimize reporter degradation from tagged peptides in TMT. The authors report an increase in reporter ion signal of 4-5-fold compared with the conventional SPS-MS3 method, and faster duty cycles. These features increase the dynamic range of proteomic analyses. The authors comment that the methodology is also compatible with real-time searching of every MS2 scan to allow triggering of quantitative MS3 only for identifiable peptides, an approach that affords yet further gains in performance.

Piga I, Koenig C, Lechner M, Sabatier P, Olsen J V. Formaldehyde fixation helps preserve the proteome state during single-cell proteomics sample processing and analysis. *Journal of Proteome Research* 24;2025:1624-1635.

Piga *et al.* show that single-cell proteomic analysis can be performed on cells in a label-free workflow after short-term formaldehyde fixation without affecting the analytical results. Their procedure employs non-ionic detergents compatible with mass spectrometry and avoids the need for high-temperature heating and sonication to recover proteins for analysis. Stabilization of single-cell proteomes in this way is anticipated to enable extensive fluorescence-activated cell sorting (FACS) and the shipment of cells to single-cell proteomics facilities for analysis, thereby improving accessibility of the technology.

## FUNCTIONAL GENOMICS & PROTEOMICS

Pacalin N M, Steinhart Z, Shi Q, Belk J A, Dorovskyi D, Kraft K, Parker K R, Shy B R, Marson A, Chang H Y. Bidirectional epigenetic editing reveals hierarchies in gene regulation. *Nature Biotechnology* 43;2025:355-368.

Hsiung C C S, Wilson C M, Sambold N A, Dai R, Chen Q, Teyssier N, Misiukiewicz S, Arab A, O’Loughlin T, Cofsky J C, Shi J, Gilbert L A. Engineered CRISPR-Cas12a for higher-order combinatorial chromatin perturbations. *Nature Biotechnology* 43;2025:369-383.

The expression of any particular gene may be regulated by large numbers of enhancers working coordinately. For want of technical approaches, however, little is presently known about the kinds of complex interactions between enhancers that comprise the dynamic regulatory network integrating the expression of many genes that ultimately control cell phenotype. Two groups describe new, combinatorial CRISPR methods for use in native, cellular genomes to help address this problem. Pacalin *et al.* developed a system that simultaneously enables the activation and repression of two distinct genes in a cell. They fuse an activator domain to catalytically dead Cas9 (dCas9) from *Staphylococcus aureus*, and fuse a repressor domain to dCas9 from *Streptococcus pyrogenes*. Directed to interact with distinct genes via distinct guide RNAs (gRNAs), these two constructs perturb transcription of the two genes in opposite directions: one activating (CRISPRa) and the other repressing (CRISPRi). Using single-cell sequencing in a Perturbseq approach, the authors employ this platform to investigate the interactions between two hematopoietic transcription factors, GATA1 and SPI1, which function antagonistically in directing precursor cells toward the erythroid or myeloid lineages. The authors discover synergistic patterns of regulation of downstream target genes. They also investigate the regulation of interleukin-2 expression in T cell activation, a study that reveals hierarchical interactions between enhancers. Hsiung *et al.* focus on multiplexing CRISPRi. They create a variant of Cas12a that enhances the stability of RNA-DNA complexes *via* DNA nicking, thereby improving CRISPRi activity. This advantage enables the authors to perform 6-plex CRISPRi for high-throughput screening in human cells, and 10-plex screening in well-based assays. They deploy this platform to study regulatory interactions between enhancers in the expression of the proto-oncogene, *Myc*. These methodologies are anticipated to contribute substantially to the understanding of gene regulatory mechanisms.

Mahendrawada L, Warfield L, Donczew R, Hahn S. Low overlap of transcription factor DNA binding and regulatory targets. *Nature* 642;2025:796-804.

Transcription factors, according to the standard conceptual model, function by binding to enhancers or promoters located a short distance upstream of their targets. Yeast has some 150 transcription factors. Mahendrawada *et al.* here map the binding locations and expression targets of 126 of them systematically to evaluate the standard model. Yeast is judged to represent a suitable model of gene regulation in higher eukaryotes: although long-range enhancers that occur in mammalian cells are unknown in yeast, transcription mechanisms appear otherwise similar. They map binding by fusing each transcription factor to micrococcal nuclease, and use chromatin endogenous cleavage with sequencing (ChEC-seq) to locate binding sites. Expression targets are identified by serial assays of changes in nascent RNA levels following rapid depletion of each transcription factor, induced by the auxin degron system. The authors’ results show that while many transcription factors conform to the standard model, many do not. The findings include limited overlap between transcription factor binding and genes that are regulated, and many regulatory targets are remote from transcription factor binding sites. Many transcription factors are further observed to behave as activators of some genes and inhibitors of others. These observations suggest that our present understanding of transcription factor function is far from adequate. Investigators interested in the function of transcription factors in higher eukaryotes will accordingly wish to interpret new results with caution.

## IMAGING

Zhang J, Zhang K, Wang K, Wang B, Zhu S, Qian H, Ma Y, Zhang M, Liu T, Chen P, Shen Y, Fu Y, Fang S, Zhang X, Zou P, Deng W, Mu Y, Chen Z. A palette of bridged bicycle-strengthened fluorophores. *Nature Methods* 22;2025:1276-1287.

The use of high-intensity lasers, prolonged exposure, and repeated imaging cycles required by super-resolution microscopy techniques renders commonly used dyes susceptible to photobleaching (photo-induced damage or degradation) and photoblueing (photoconversion to a blue-shifted molecule). Addressing these issues while also retaining fluorophore brightness, water solubility, and biological membrane permeability has proven difficult. Chen *et al.* here describe a new class of fluorophores that fulfill all these diverse requirements. They utilize azabicyclo[3.2.1]octane auxochromes (moieties that modify the chromophore’s ability to absorb light), and, within these auxochromes, incorporate the solubility-enhancing substituents SO2- or O-. The structure of their dyes resists photobleaching and photobluing by minimizing the formation of radical-cations or iminium intermediates during photo-oxidation. The dyes provide a broad spectral range for application in fluorescence imaging.

## MACROMOLECULAR SYNTHESIS & SYNTHETIC BIOLOGY

Wiegand D J, Rittichier J, Meyer E, Lee H, Conway N J, Ahlstedt D, Yurtsever Z, Rainone D, Kuru E, Church G M. Template-independent enzymatic synthesis of RNA oligonucleotides. *Nature Biotechnology* 43;2025:762-772.

Synthetic RNA oligonucleotides have become exceedingly important therapeutic agents, and their uses continue to expand rapidly. The solid-phase phosphoramidite method presently employed for their synthesis has limited scalability, utilizes large quantities of flammable, toxic organic solvents, and requires special production of modified nucleotide components for therapeutic efficacy. Template-independent enzymatic methods, of proven effectiveness for DNA synthesis, but not hitherto for RNA synthesis, are theoretically attractive because of their potential to dispense with organic solvents, synthesize long oligonucleotides, and readily incorporate modified nucleotides. Wiegland *et al.* here make substantial progress toward enzymatic methodology fulfilling these promises. They deploy an engineered poly(U) polymerase for the purpose. This rapidly incorporates nucleotides, either native and selectively modified, into the growing chain. The nucleotides are initially protected with a 3′-*O*-allyl ether to limit chain extension. The blocking group is removed by a mild deal-lylation reaction prior to addition of the next nucleotide. The method may be executed either in solution or on a solid support. With further modification of the polymerase to improve coupling efficiency (presently averaging 95%), and shortening of the deprotection step, this methodology is anticipated to contribute significantly to future RNA oligonucleotide manufacturing.

Witte I P, Lampe G D, Eitzinger S, Miller S M, Berríos K N, McElroy A N, King R T, Stringham O G, Gelsinger D R, Vo P L H, Chen A T, Tolar J, Osborn M J, Sternberg S H, Liu D R. Programmable gene insertion in human cells with a laboratory-evolved CRISPR-associated transposase. *Science* 388;2025:eadt5199.

Witte *et al.* demonstrate programmable genomic integration of kilobase-size DNA cargos into human cells using a CRISPR-associated transposase. CRISPR-associated transposases use nuclease-deficient CRISPR machinery to insert DNA into locations specified by a guide RNA, without creating double-stranded DNA breaks. Transposases have hitherto worked with low efficiency in human cells, but the authors here employ iterative rounds of phage-assisted continuous evolution to select variants of the transposase TnsB with greatly enhanced integration activity. When combined with other engineered CRISPR-associated components, the authors achieve 10-30% integration efficiency in human cells with cargos of greater than 10 kb. The results encourage optimism for the development of therapeutic capabilities to deliver intact genes for rescue of function in loss-of-function diseases and to produce cells for cancer immunotherapy, among other applications.

Hua Y, Tay N E S, Ye X, Owen J A, Liu H, Thompson R E, Muir T W. Protein editing using a coordinated transposition reaction. *Science* 388;2025:68-74.

Beyer J N, Serebrenik Y V, Toy K, Najar M A, Feierman E, Raniszewski N R, Korb E, Shalem O, Burslem G M. Intracellular protein editing enables incorporation of noncanonical residues in endogenous proteins. *Science* 388;2025:eadr5499.

Two groups demonstrate editing the sequence of a native, folded protein by insertion of a peptide within the protein’s primary sequence. They do so without disrupting the recipient protein’s function, and perform the editing procedure under conditions suitable for use within a live cell. Both groups approach their task using split inteins. An intein is a peptide segment that catalyzes its own excision from a protein sequence, reforming a native peptide bond between the remaining flanking sequences. Both groups employ two pairs of fast-reacting split inteins. An exchangeable cargo segment flanked by a pair of split inteins interacts with a recipient protein bearing within its sequence matching intein acceptors flanking the acceptor site. Interaction between the donor and acceptor results in rapid exchange of the cargo segment between donor and recipient: a transposition reaction. The cargo can be engineered by either genetic code expansion or solid-phase peptide synthesis to incorporate non-canonical amino acids, reactive species, fluorophores, affinity tags, or acceptor sites for post-translational modification. Cargo peptides may be delivered to cells by electroporation or *via* lipid nanoparticles. This technology opens new possibilities for the study of cell signaling, protein interaction, and regulation by means of protein editing within live cells. It also enables drug discovery and protein design by protein sectional synthesis.

